# Potential roles of nitrate and nitrite in nitric oxide metabolism in the eye

**DOI:** 10.1038/s41598-020-69272-9

**Published:** 2020-08-05

**Authors:** Ji Won Park, Barbora Piknova, Audrey Jenkins, David Hellinga, Leonard M. Parver, Alan N. Schechter

**Affiliations:** 1 Molecular Medicine Branch, National Institute of Diabetes and Digestive and Kidney Diseases, National Institutes of Health, 10 Center Drive, 9N314, Bethesda, MD 20892 USA; 2 MedStar Health Research Institute, Washington, DC USA; 3 Department of Ophthalmology, MedStar Georgetown University Hospital, Washington, DC USA

**Keywords:** Biochemistry, Physiology, Medical research

## Abstract

Nitric oxide (NO) signaling has been studied in the eye, including in the pathophysiology of some eye diseases. While NO production by nitric oxide synthase (NOS) enzymes in the eye has been characterized, the more recently described pathways of NO generation by nitrate (NO_3_^−^) and nitrite (NO_2_^−^) ions reduction has received much less attention. To elucidate the potential roles of these pathways, we analyzed nitrate and nitrite levels in components of the eye and lacrimal glands, primarily in porcine samples. Nitrate and nitrite levels were higher in cornea than in other eye parts, while lens contained the least amounts. Lacrimal glands exhibited much higher levels of both ions compared to other organs, such as liver and skeletal muscle, and even to salivary glands which are known to concentrate these ions. Western blotting showed expression of sialin, a known nitrate transporter, in the lacrimal glands and other eye components, and also xanthine oxidoreductase, a nitrate and nitrite reductase, in cornea and sclera. Cornea and sclera homogenates possessed a measurable amount of nitrate reduction activity. These results suggest that nitrate ions are concentrated in the lacrimal glands by sialin and can be secreted into eye components via tears and then reduced to nitrite and NO, thereby being an important source of NO in the eye.

## Introduction

The NO generation pathway from L-arginine by endogenous NOS enzymes under normoxic conditions has been central in identifying the physiological roles of NO in numerous biological processes^1^. Considering the half-life of NO is only 2 ms in blood and less than 2 s in tissues^2,3^, stabilization of NO to nitrite and nitrate can be a good storage method for bioavailable NO since these anions are able to be reduced back to NO under hypoxic conditions. It has been shown that nitrate absorbed from dietary sources can be converted to nitrite, mainly by commensal bacterial nitrate reductases in the oral cavity after concentration from the blood by the salivary glands^4^. Nitrite in tissues can be reduced to NO by several pathways such as deoxyhemoglobin^5^^,^ deoxymyoglobin^6,7^, molybdenum containing enzymes, mainly XOR^8^ and non-enzymatic mechanism in a low pH environment^9,10^ or ascorbic acid^11^. The significance of these serial reduction pathways in NO generation has recently gained great attention. Many studies show that nitrate/nitrite supplementation exhibits beneficial effects in cardiovascular system, especially where NOS-derived NO generation is impaired, as well as improving some exercise performance measures^12^. The infusion of nitrite ions showed an increase in forearm blood flow and a decrease in mean arterial pressure in healthy humans^5,13^. A nitrate (NaNO_3_) supplementation study also showed a reduction in diastolic blood pressure in healthy volunteers^14^ and later these nitrate effects in blood pressure regulation were reported to be dependent on the oral microbiome^15^. Several other clinical studies have followed and demonstrated the contribution of the nitrate–nitrite–NO pathway to many physiological and pathophysiological conditions^12,16^.

In 1990s, it was suggested that NO is also involved in the regulation of ocular blood flow^17,18^ and NOS3 alteration was thought to be a contributing factor to glaucoma development^19–21^. The balance between production and elimination of aqueous humor is critical in controlling ocular pressure and the modulation of conventional aqueous humor outflow through the trabecular meshwork and Schlemm’s canal mediated by NO is now a target for glaucoma therapy^22^. Recently, the FDA approved an NO-donating prostaglandin analogue, latanoprostene bunod, for reduction of intraocular pressure in patients with open-angle glaucoma or ocular hypertension^23^ and the clinical importance of NO signaling in the eye has been getting more attention. However, there are not many reports on the effects of nitrate and nitrite as NO precursors in eye physiology. A recent paper showed an association of dietary nitrate intake with primary open-angle glaucoma suggesting a higher nitrate consumption lowers the risk of getting a primary open-angle glaucoma by 20–30%^24^. And another study suggested an association between dietary nitrate intake and incidence of age-related macular degeneration^25^.

To study the potential roles of the nitrate–nitrite–NO pathway in normal eye physiology, we analyzed nitrate and nitrite levels in components of the eye and lacrimal glands in porcine samples. Lacrimal glands, located in the upper lateral region of each orbit, produce the aqueous portion of the tear film and share similarities with salivary glands in developmental processes^26^. Since it is known that nitrate is concentrated in salivary glands from blood and secreted into the oral cavity where it is reduced to nitrite by commensal oral bacteria^27^^,^ we hypothesized that nitrate levels in the lacrimal glands would be also higher than other organs. The nitrate ions might then be secreted from the lacrimal glands into tears and reduced to nitrite by endogenous enzymes or the eye microbiome on the ocular surfaces, thereby functioning as NO sources in the eye.

Our results show that lacrimal glands exhibited much higher levels of nitrate compared to salivary glands and other organs such as liver and skeletal muscle. In addition, Western blotting analyses confirmed the expression of sialin in the lacrimal glands and other eye components. XOR protein was present in cornea and sclera tissue and we confirmed the nitrate reduction activity of sclera and cornea homogenates. These results suggest that NO generation via the nitrate–nitrite–NO pathway may contribute to physiological and pathophysiological processes in the eye.

## Results

### Nitrate and nitrite contents in eye components

**Figure 1:**
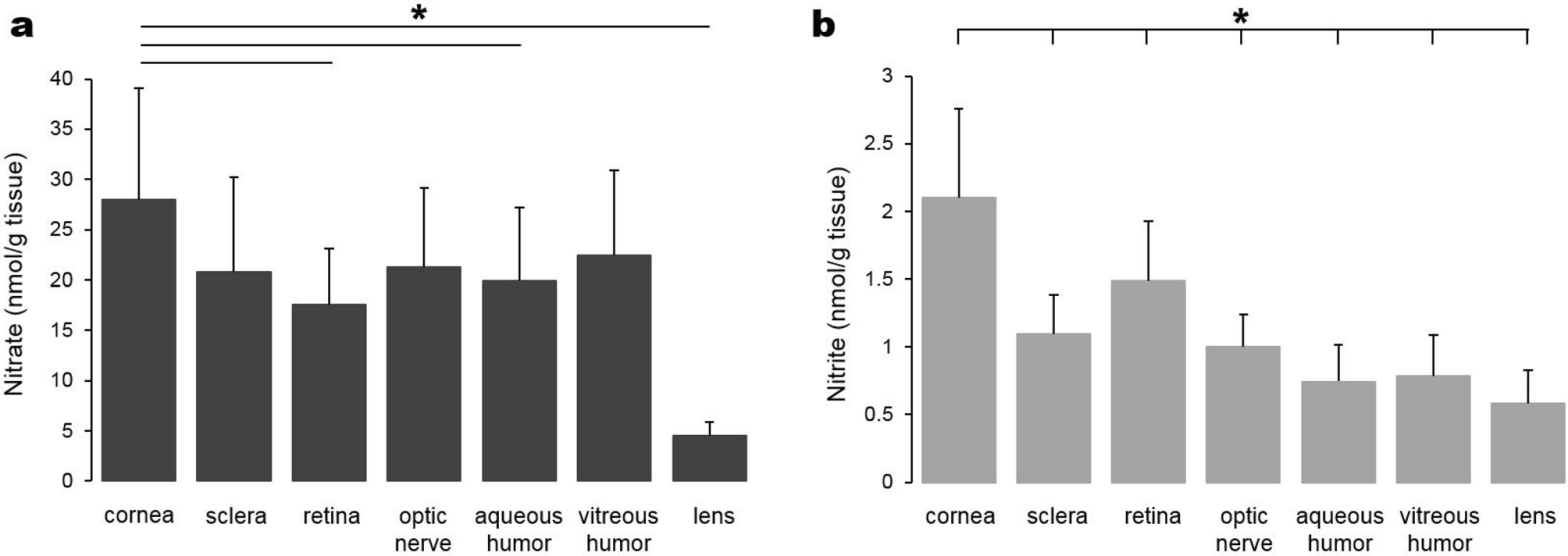
Nitrate and nitrite contents in porcine eye components. Porcine eyes were dissected into cornea, sclera, retina, optic nerve, aqueous and vitreous humor and lens. Each eye component was weighed and homogenized, then deprotonated by centrifugation after the addition of methanol. The supernatant was used for nitrate (**a**) and nitrite (**b**) ion content measurement using a standard chemiluminescence method with vanadium chloride or tri-iodide solution respectively, n = 9–16 in each component (*p < 0.05, comparison between cornea and other individual tissue).

To examine the levels of nitrate and nitrite ions in each eye component, we dissected porcine eyes into cornea, lens, retina, sclera, optic nerve, aqueous and vitreous humor (Fig. 1). Under these basal conditions and among these eye components, the cornea appears to contain the highest levels of nitrate and nitrite ions (28 ± 11 and 2.1 ± 0.7 nmol/g respectively) while lens had the least amount of nitrate and nitrite (4.5 ± 1.3 and 0.58 ± 0.2 nmol/g respectively). The amounts of nitrate ions in the other eye parts studied were similar to each other, ranging from 18 to 22 nmol/g. The aqueous and vitreous humor contained slightly less nitrite ions compared to retina, sclera and optic nerve tissues.

### Nitrate and nitrite levels in lacrimal and salivary glands and fluids from these glands

**Figure 2:**
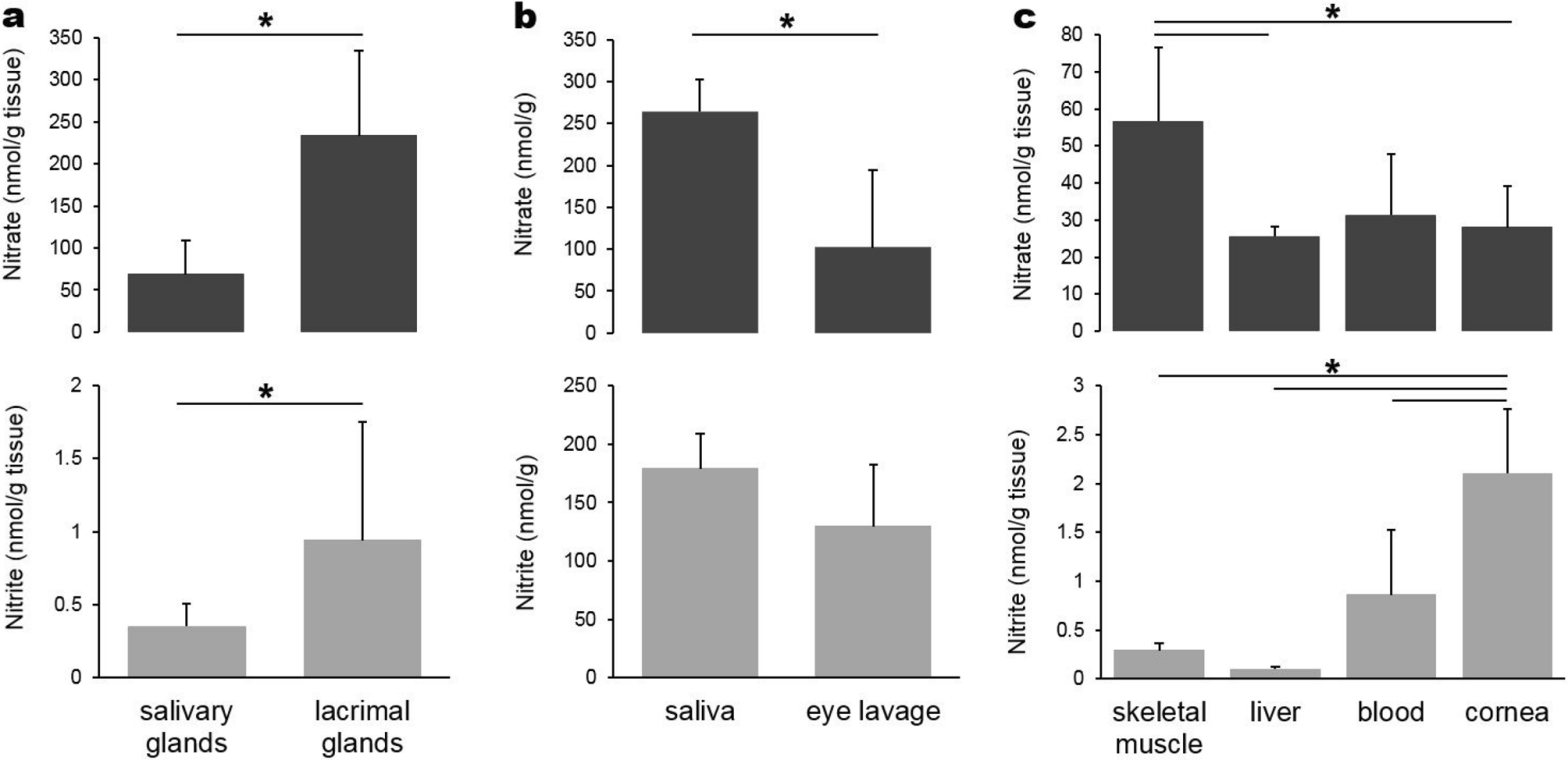
Measurement of nitrate and nitrite levels in porcine tissues. Salivary (combination of parotid, submandibular and sublingual glands) and lacrimal glands (**a**), saliva and eye lavage (**b**) and other internal tissues and cornea (**c**) were analyzed for nitrate and nitrite contents using a standard chemiluminescence method with vanadium chloride or tri-iodide solution respectively, n = 4–17 in each tissue (*p < 0.05).

Nitrate ions are now known to be concentrated in salivary glands from the blood and excreted into saliva, then reduced to nitrite by oral bacteria^28,29^. We hypothesized that lacrimal glands might contain high levels of nitrate as with the salivary glands, based on the similarities between those glands as the major exocrine glands of the head. We analyzed nitrate and nitrite levels in porcine lacrimal and salivary glands as well as eye lavage sample and saliva (Fig. 2). Three different salivary glands (parotid, submandibular and sublingual glands) were collected and the nitrate levels for parotid, submandibular and sublingual glands were 82.5 ± 50.5, 55.3 ± 24.3 and 52.2 ± 40.0 nmol/g respectively. Nitrite levels for the parotid, submandibular and sublingual glands were 0.38 ± 0.06, 0.23 ± 0.12 and 0.44 ± 0.17 nmol/g respectively. The values from these three glands were averaged for nitrate and nitrite analyses in Fig. 2a. Surprisingly, nitrate levels in lacrimal gland were much higher than those in salivary glands (234 ± 101 vs 68 ± 40 nmol/g). However, fluids from these glands, eye lavage (closest to representative of tears) and saliva respectively, showed the opposite trend in nitrate and nitrite levels (Fig. 2b), higher nitrate and nitrite contents in saliva than in eye lavage (264 ± 38 vs 102 ± 92 for nitrate, 179 ± 30 vs 130 ± 53 for nirite). Unfortunately, due to technical reasons, we could not measure concentration in genuine tear samples. For comparison, human salivary nitrate has been reported as 204 to 302 nmol/g^30^ and human tear nitrate was reported as 20–109 μM^31,32^. We also analyzed nitrate and nitrite levels in some of non-ocular tissues to compare them with cornea, a representative ocular tissue (Fig. 2c). We previously demonstrated that skeletal muscle is the major nitrate storage organ and there is a nitrate gradient between skeletal muscle and blood and liver^33^. Figure 2c shows that cornea contains similar nitrate amount to liver, but lesser amounts than skeletal muscle, while nitrite levels were higher in cornea than other non-ocular tissues examined in this study.

### Protein expression levels in porcine tissues and human ocular cell lysates

**Figure 3:**
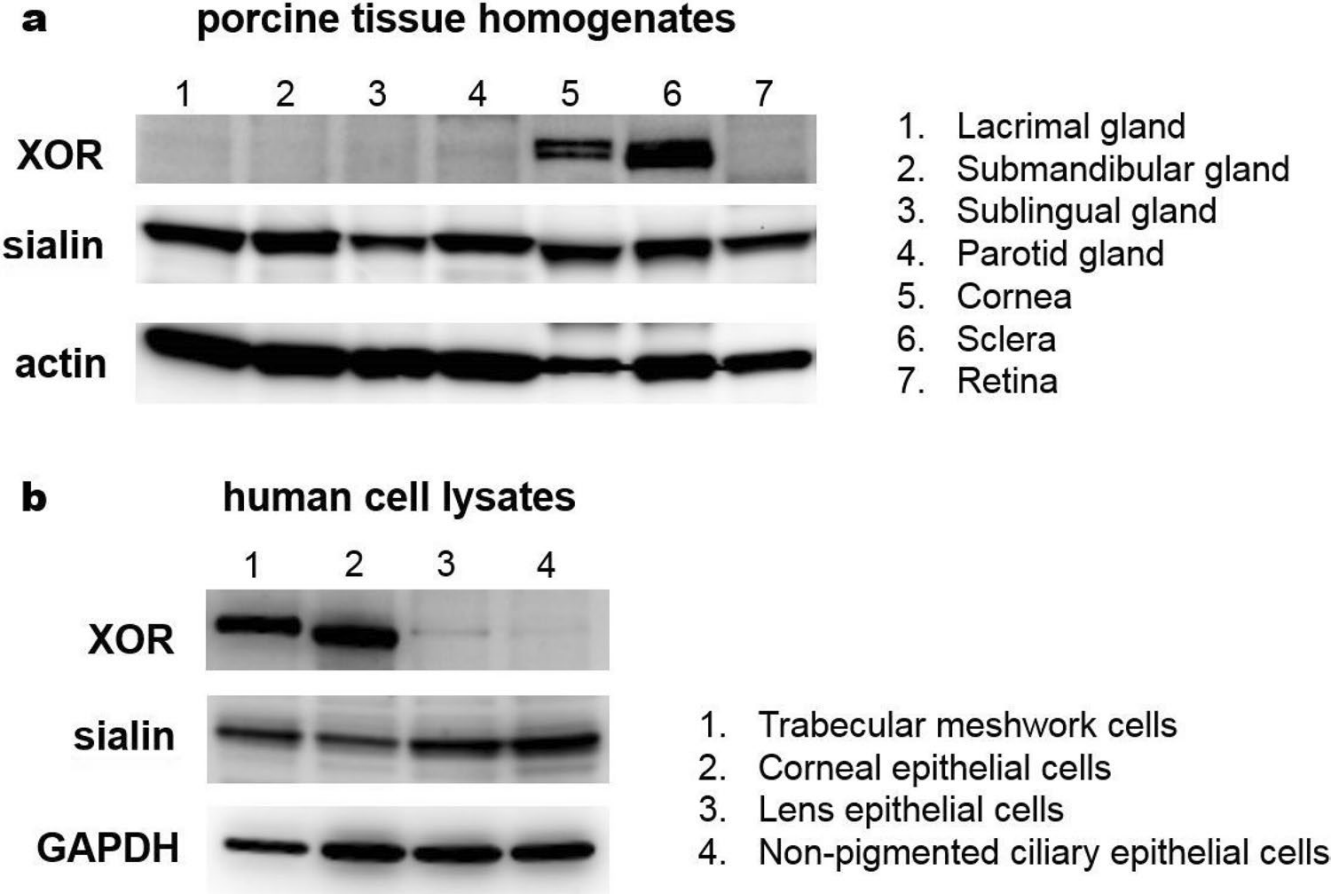
Analyses of nitrate metabolism-related proteins by Western blotting. Protein samples (50 μg) were run on SDS-PAGE and transferred onto nitrocellulose membranes for immunoblotting. Primary antibodies (anti-XOR, -sialin, -actin and -GAPDH) were incubated overnight at 4 °C and then secondary antibodies conjugated with horseradish peroxidase were used for the enhanced chemiluminescence detection. The uncropped image of the Western blot is presented on Supplementary information.

To evaluate the levels of proteins possibly involved in the nitrate metabolism pathway, we performed Western blotting using porcine eye and gland tissue homogenates and several human ocular cell lysates (Fig. 3). A nitrate transporter protein, sialin, was highly expressed in both lacrimal and salivary glands as well as in other porcine eye components such as cornea, sclera and retina. A mammalian nitrate/nitrite reductase, XOR was not detected in the gland samples, but was present in porcine cornea and sclera (Fig. 3a). XOR was also detected in human trabecular meshwork and corneal epithelial cell lysates, which is consistent with the porcine result of XOR expression. Sialin protein was present in four different human ocular cell lysates examined (Fig. 3b).

### Nitrate reduction activity in sclera and cornea homogenates

**Figure 4:**
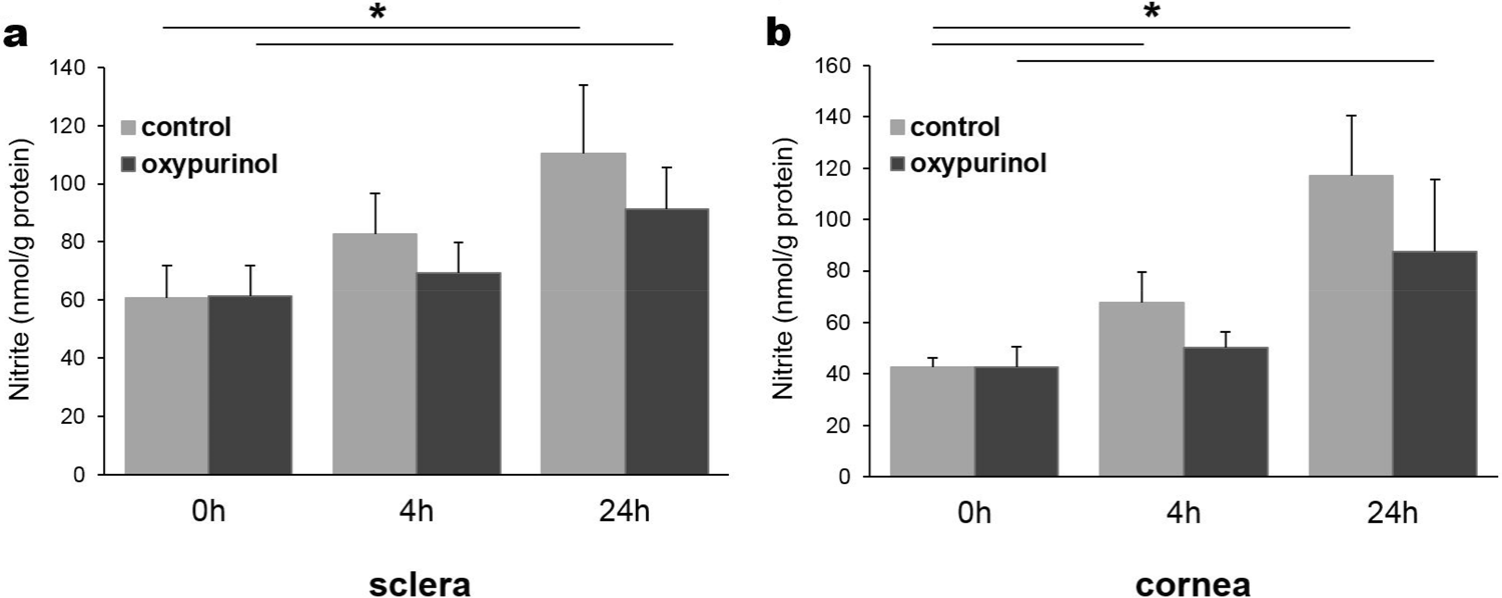
Nitrate reduction activity in sclera and cornea homogenates. Sclera (**a**) and cornea (**b**) homogenate was mixed with cofactors such as NADPH, UDP glucuronic acid, NAD^+^, NADH and glutathione with or without oxypurinol (200 μM). After the addition of 500 μM nitrate, homogenate mixture was incubated at 37 °C under the atmosphere containing 2% oxygen and aliquots were taken at 0, 4 and 24 h and analyzed by chemiluminescence to determine nitrite generation (n = 4 for sclera, n = 5–7 for cornea, *p < 0.05).

Since we confirmed the expression of XOR protein in sclera and cornea, we tested whether nitrate conversion to nitrite occurs in these eye tissues. Both sclera (Fig. 4a) and cornea (Fig. 4b) showed increased nitrite levels after the addition of sodium nitrate for 4 and 24 h at 2% oxygen. This nitrate reduction activity was only slightly reduced in the presence of an XOR inhibitor, oxypurinol, both in sclera and cornea which may suggest that there are also XOR-independent nitrate reduction pathways in these eye tissues.

In our previous publications^33,34^, we used an established nitrate reduction activity assay using tissue homogenates with protein concentrations at 5–7 mg/ml. However, the fact that both sclera and cornea are dense tissues which mostly consist of collagen fibrils^35^ made tissue homogenization quite challenging and in most cases we were unable to obtain highly concentrated protein solutions. Therefore 2.5 mg/ml protein for both sclera and cornea homogenates was used for the assays. It should be noted that the lower than usual protein concentration and longer time required for its extraction could have deleterious effects on nitrate reductases present in these tissues.

## Discussion

The NO signaling pathway has been known to be involved in various physiological processes in the eye, especially in regulating ocular blood flow and intraocular pressures. The fact that the FDA recently approved an ophthalmic solution, latanoprostene bunod, as a source of NO and latanoprost acid for open-angle glaucoma and ocular hypertension emphasized the clinical significance of NO signaling in the eye^22,23^. The roles of endogenous NOS enzymes for regulation of intraocular pressure and blood flow have been studied in the eyes of many species since the discovery of NO and NOS pathway^36^ while the alternative more recently discovered NO generation mechanisms, which involves nitrate/nitrite reduction to NO, have still not gained much focus in the eye research field.

It is important to note that the endogenous NOS pathway becomes inactive under hypoxic situations since NOS enzymes require oxygen for its activity in NO production; on the other hand, the nitrate/nitrite reduction pathways are enhanced under hypoxia making them good backup systems for the shutdown of oxygen-dependent NOS activity. Moreover, a human diet rich in vegetables (app. 300 g) is known to provide more nitrate than what is produced by all three NOS enzymes endogenously over a day^37^. Nitrate absorbed from diets can go through stepwise reduction pathways starting from the action of oral bacteria^4^. In addition to commensal bacteria, the presence of functional mammalian nitrate reductases has been also reported and studies showed that the inhibition of XOR attenuated nitrate reduction activity in rodent and human tissues^33,34,38^. Furthermore, it has been reported that even germ free mice showed some extent of nitrate reduction activity^39^. Considering the relatively hypoxic environment in the eye (the mean pO_2_ of the cornea-24.1 mmHg, the anterior chamber angle-12.9 mmHg)^40^^,^ it is reasonable to hypothesize that the nitrate/nitrite/NO pathway exists in this organ and that the reductive mechanism could contribute to NO-mediated physiological processes in the eye.

It is known that the salivary glands in several species are able to concentrate nitrate from the circulation and excrete it to the mouth saliva where oral commensal bacteria can reduce it to nitrite. In the eye, there is a similar exocrine gland, the lacrimal gland, which produces important tear components to help protect ocular surfaces. It has been reported that human tears contain relatively high concentrations of nitrate 20–109 μM^31,32^ and we hypothesized that lacrimal glands would have similar functions to salivary glands in nitrate metabolism. Our results from porcine tissues show that the lacrimal gland itself contains the most nitrate (234 ± 101 nmol/g) compared to other organs examined, including salivary glands. Due to the difficulties in collecting basal tears from anesthetized animals, we collected eye lavage samples and its nitrate level was about 102 ± 92 nmol/g. Since we cannot precisely calculate the dilution factors in the eye lavage samples, it is not possible to get exact numbers for tear nitrate levels. However, it is likely that tears contain higher nitrate levels than internal organs since eye lavage samples contain much higher levels of nitrate than other organs such as liver and skeletal muscle (Fig. 2b, c).

We also measured nitrate levels in several eye components and the results show that cornea contains slightly higher nitrate levels than other internal eye parts, and lens contains the least amount of nitrate among the eye tissues we studied (Fig. 1a). This nitrate gradient between cornea and lens may be explained by our hypothesis that tears contribute to nitrate distribution to the eye and nitrate can be then utilized in the eye tissues such as cornea. This is supported by the fact that cornea contains more nitrite than other eye components (Fig. 1b). In addition, we found remarkably high levels of nitrite in eye lavage (130 ± 53 nmol/g) and saliva (179 ± 30 nmol/g) samples while other internal organs contain less than 1 nmol/g of nitrite (Fig. 2b, c). These results suggest that nitrate present in high levels in both lacrimal and salivary gland was metabolized to nitrite and then secreted into tears and saliva respectively.

Another important finding in our study is that we confirmed the expression of a nitrate transporter, sialin, in eye components and lacrimal glands (Fig. 3a). Sialin is known to play a major role in nitrate transport in salivary glands^41^ and to our knowledge, our current study is the first to show the presence of sialin in lacrimal gland and in eye components. In our previous study with skeletal muscle cells, we showed that the inhibition of anion transporters or knock-down of sialin significantly decreased the amount of nitrates taken up by muscle cells^42^ confirming a crucial role of sialin in nitrate uptake in various tissues. We show in the present study by Western blotting analyses that the expression level of sialin in lacrimal gland is comparable to that in salivary glands. This result suggests that the high levels of nitrate we observed in lacrimal gland and eye lavage sample are at least partly mediated by the action of sialin. We also observed the expression of a mammalian nitrate/nitrite reductase protein, XOR in sclera and cornea tissues (Fig. 3a) which suggests a possibility of a functional nitrate reduction pathway in eye tissues. Importantly, we also confirmed the expression of XOR and sialin in human trabecular meshwork and corneal epithelial cells (Fig. 3b). Trabecular meshwork is a sieve-like structure of the limbal sclera and responsible for draining the aqueous humor, thereby playing a critical role in ocular pressure regulation. Thus, existence of the nitrate–nitrite–NO pathway in trabecular meshwork would have a beneficial effect in modulating ocular pressure. More mechanistic studies on the nitrate/nitrite reduction using trabelular meshwork cells will provide a better insight into the NOS-independent NO pathways in the ocular system. In addition, we were able to measure the nitrate reduction activity in sclera and cornea tissue homogenates (Fig. 4) although the activity was lower than other organs used in our previous studies^33,34^. It is also possible that eye microbiome could participate in nitrate reduction on ocular surfaces, but the current study was not designed to measure the bacterial contribution to nitrate metabolism. Both bacterial enzyme-mediated and mammalian enzyme-mediated nitrate reduction pathways will probably play a part in the NO signaling pathway in the eye.

**Figure 5:**
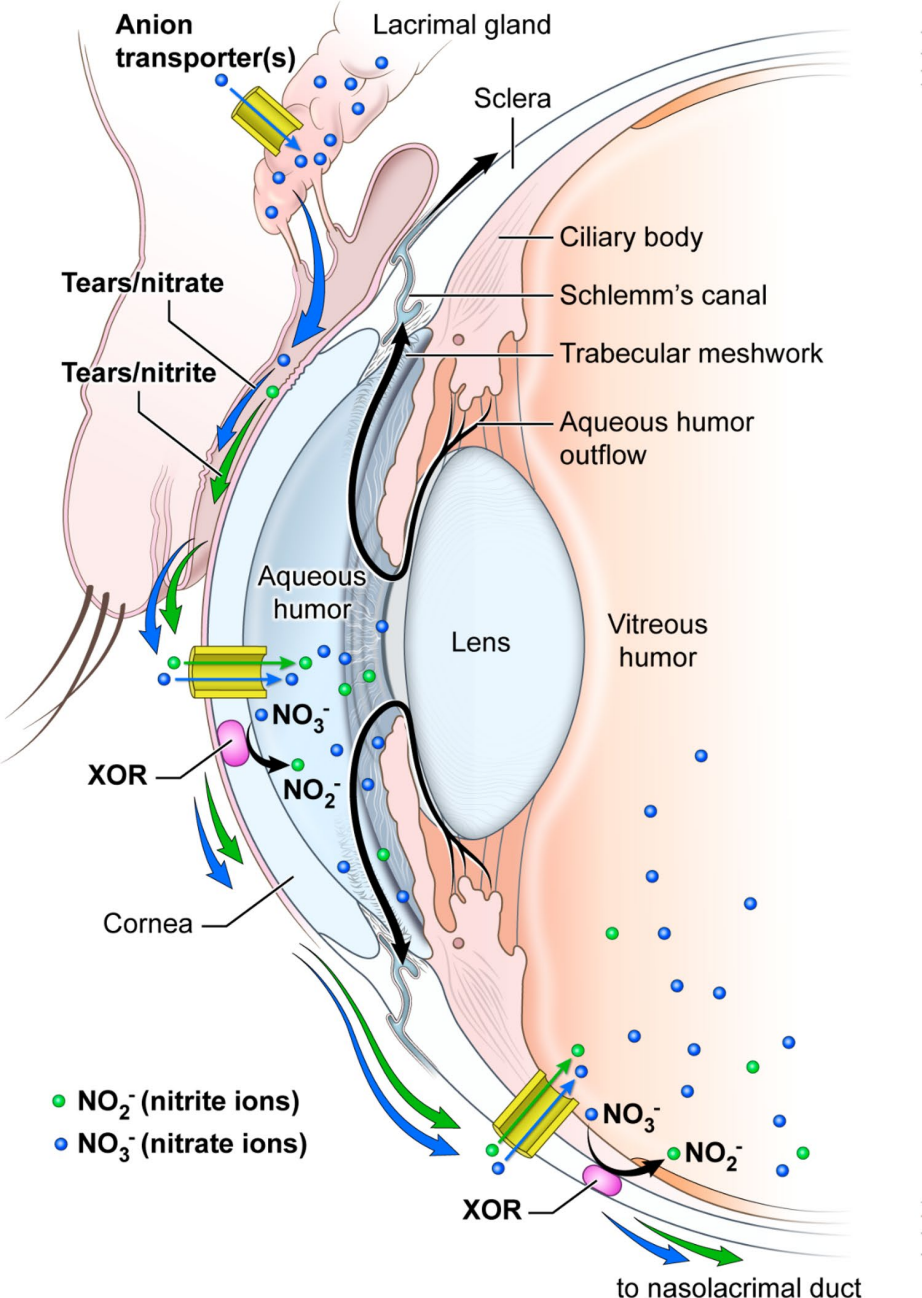
A diagram of the proposed major pathways for nitrate ion transport and metabolism in the anterior structures of the human eye. We present a schematic of the structure of the anterior part of the human eye with our hypotheses about possible transport of nitrate (NO_3_^−^) and nitrite (NO_2_^−^) ions and their metabolism by reductive chemistry. We postulate that the lacrimal glands act similarly to salivary glands by concentrating nitrate from the blood and secreting it into ducts which transport these ions in the tear fluids to the conjunctiva, where bacterial enzymes can reduce them to nitrite and NO. We have shown that majority of eye tissues express sialin, which is a known nitrate ion transporter, but there may be other anion transporter molecules which can also act as nitrate transporters. These processes for formation of NO in the eye are in addition to its formation from arginine by the NOS enzymes that is prevalent at normoxia that has largely been studied up until now (not shown on the diagram). Nitrate and nitrite ions can also enter the sclera and cornea where xanthine oxidoreductase (XOR) and other as yet unknown agents appear to be able to reduce them into nitrite and NO, especially under hypoxic conditions, and then diffuse into the anterior chamber of the eye. This would likely result in increased flow of intra-ocular fluids into Schlemm’s canal, as well as increased blood flow, especially in the retina. The tear fluid is also collected into the naso-lacrimal ducts which flow into the nasal cavity and may contribute to the high levels of exhaled NO from the oropharyngeal cavity. Symbols: blue small circles: nitrate ions; green small circles: nitrite ions; blue arrows: flow of nitrate ions; green arrows: flow of nitrite ions; yellow half cylinders: anion transporter membrane proteins, including sialin.

The diagram shown in Fig. 5 is our suggested view of the major nitrate metabolism pathways in the eye based on the data presented in this paper (see figure legend). In conclusion, these results suggest that the nitrate/nitrite/NO reduction pathway in the eye may play an important physiological role for NO homeostasis. Although NO is known to reduce intraocular pressure and improve blood flow in the eye, the importance of the nitrate–nitrite–NO pathway has not been fully appreciated in these aspects of eye physiology. Understanding the role of this reductive pathway and its implications in ocular diseases may also open new therapeutic modalities. Given that diet is the major source of available nitrate, it may be a promising and easy intervention to supplement nitrate through a nitrate rich diet in susceptible eye diseases. However, more detailed mechanistic studies are required to understand better the rates of nitrate and nitrite ion metabolism in eye tissues. Clinical studies with normal volunteers and patients with various eye diseases are necessary to determine the relvance of these findings in the pig model to normal and abnormal human eye physiology.

## Material and methods

### Animal study

This research was conducted as part of animal protocols approved by the IACUC (Institutional Animal Care and Use Committee) of Medstar Health Research Institute in compliance with the Animal Welfare Act and the Guide for the Care and Use of Laboratory Animals, 8^th^ ed. Both male and female Yorkshire domestic cross swine weighing between 35–65 kg and sourced from Thomas D. Morris, Inc. (Reisterstown, MD) were used. On arrival, animals were acclimated for a minimum of 72 h and housed in an AAALAC (Association for Assessment and Accreditation of Laboratory Animal Care)-accredited facility with environmental enrichment. Animals were fed twice daily with a commercial chow (Teklad miniswine diet, 8753C, Envigo, Madison, WI). Fresh water was provided ad libitum by an automated system. All tissue samples were obtained from animals undergoing acute procedures and euthanized according to facility approved procedures. Euthanasia was accomplished with a single intravenous injection of saturated potassium chloride (4.6 ml per 10 kg body weight, IV bolus, using a 4.2 M KCl concentration) while the animals were maintained on isoflurane gas anesthesia per AVMA (American Veterinary Medical Association) guidelines. Animal expiration was confirmed until no visible respirations (apnea) were noted and readings of zero for the heart rate, pO_2_ level and CO_2_ saturation were noted. After collecting gland samples, histological examination was performed to confirm the target tissue.

### Sample preparation for nitrate and nitrite measurements

Standard chemiluminescence assays for measuring nitrite and nitrate contents were performed according to previously published protocols^43,44^. Blood was drawn from the femoral artery into vacutainer tubes containing sodium citrate (Becton Dickinson, Franklin Lakes, NJ) and immediately mixed with nitrite preserving solution (1:4) containing potassium ferricyanide, N-ethylmaleimide and NP40. Saliva was collected from the cotton gauze that was chewed by individual swine. Due to very limited volume of basal tears in anesthetized animals, eye lavage samples were prepared by sprinkling 100 µl of PBS onto the eye surface, then collecting PBS from the inner corner of the eye near the caruncula lacrimalis. Tissue samples were weighed and homogenized in 1% NP40 solution using GentleMacs (Miltenyi Biotec Inc, Auburn, CA). Proteins from all samples were precipitated by adding methanol (dilution 1:1) and subsequent centrifugation at 11,000 g for 15 min at 4 °C. Supernatants were used to determine nitrite and nitrate contents by chemiluminescence (Sievers 280i Nitric Oxide Analyzer, GE Analytical Instruments).

### Western blot analysis

Western blotting was performed to analyze the levels of XOR, sialin, actin and GAPDH protein in the eye components and lacrimal and salivary glands. Tissues were homogenized in RIPA buffer (Sigma, R0278) containing protease inhibitors (CalBiochem, 539134) and protein concentration was measured by bicinchoninic acid (BCA) assay (Thermo Scientific, 23227). Human cell lysates were purchased from ScienCell Research Laboratories (trabecular meshwork cell lysate, 6596; corneal epithelial cell lysate, 6516; lens epithelial cell lysate, 6556; non-pigment ciliary epithelial cell lysate, 6586). Denatured samples (50 µg) were run on SDS-PAGE and then transferred to nitrocellulose membranes. The membranes were incubated with primary antibodies (Anti-sialin: Alpha Diagnostics, SIAL11-A; Anti-XOR: Abcam, ab133268; Anti-pan-actin: Cell Signaling, 4968;Anti-GAPDH: Cell Signaling, 97166S) overnight at 4 °C. Goat-anti-mouse or goat-anti-rabbit antibodies conjugated with horseradish peroxidase (Jackson Immunoresearch, 115-035-003, 111-035-003) were used as secondary antibodies and followed by enhanced chemiluminescence (ECL) detection (Thermo Scientific, 34095).

### Nitrate reduction activity assay

Nitrate reduction activity assay was performed as previously described^33,34^. Briefly, cornea and sclera tissues were homogenized using GentleMacs and protein content was measured by BCA assay and adjusted to 2.5 mg/ml. The cofactor mixture with or without oxypurinol (200 μM) was added to homogenate solution and then 500 µM nitrate was added to the reaction tube lastly. The aliquots were taken at 0, 4 and 24 h and then analyzed by chemiluminescence for nitrite content. Experiments were performed at 37 °C under 2% oxygen in 100 mM phosphate buffer, pH6.5 to mimic the relatively hypoxic environment in the eye^40^.

### Statistical analysis

Values represent means ± standard deviation. Statistical significance of results was tested using the one-way ANOVA.

## Supplementary information

Supplementary Information.
