# Supplementary material for: Potential roles of nitrate and nitrite in nitric oxide metabolism in the eye
Source: Sci Rep. 2020 Aug 5;10:13166. doi: 10.1038/s41598-020-69272-9 (PMC7406513; doi:10.1038/s41598-020-69272-9)

**Supplementary Information**

**Potential roles of nitrate and nitrite in nitric oxide metabolism in the eye**

Ji Won Park^1^, Barbora Piknova^1^, Audrey Jenkins^2^, David Hellinga^2^, Leonard M. Parver^3^, and Alan N. Schechter^1^*

^1^ Molecular Medicine Branch, National Institute of Diabetes and Digestive and Kidney Diseases, National Institutes of Health, Bethesda, MD; ^2^ MedStar Health Research Institute, Washington, DC; ^3^ Department of Ophthalmology, MedStar Georgetown University Hospital, Washington, DC.

** Correspondence*: Alan N. Schechter, MD, Molecular Medicine Branch, National Institute of Diabetes and Digestive and Kidney Diseases, National Institutes of Health, 10 Center Drive, 9N314, Bethesda, MD20892; telephone: 301-496-5408; fax: 301-402-0101; email: [aschecht@helix.nih.gov](mailto:aschecht@helix.nih.gov)

The uncropped original Western blot images of Fig.3


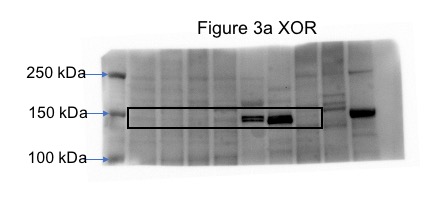


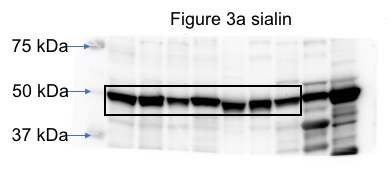


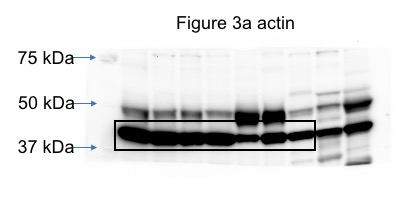


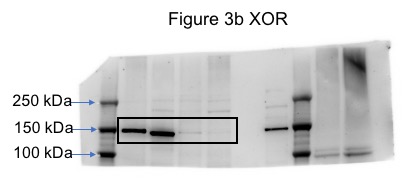


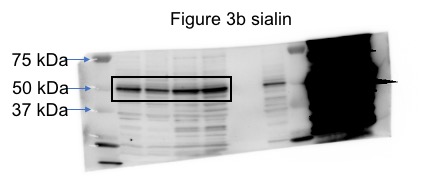


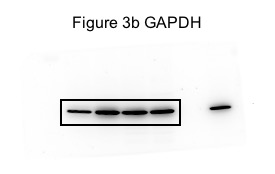

Supplement: Supplementary file 1 — Supplementary Information. [file 41598_2020_69272_MOESM1_ESM.docx]
